# Editorial: “Transcellular Cycles Underlying Neurotransmission”

**DOI:** 10.3389/fnut.2015.00018

**Published:** 2015-06-29

**Authors:** Sebastián Cerdan, Blanca Lizarbe

**Affiliations:** ^1^Instituto Investigaciones Biomédicas “Alberto Sols” CSIC-UAM, Madrid, Spain; ^2^Laboratory for Functional and Metabolic Imaging (LIFMET), SB IPSB, École Polytechnique Fédéral de Lausanne, Lausanne, Switzerland

**Keywords:** magnetic resonance imaging, magnetic resonance spectroscopy, glutamate–glutamine cycle, neuroglial interactions, neurovascular coupling

Neuronal action potentials and neurotransmitter releases induce important alterations in the extracellular *millieu*, including increased K^+^ concentrations from membrane repolarization and increased neurotransmitter levels from trans-synaptic signaling (1, 2). It becomes crucial then to remove, fast and efficiently, these ionic and neurotransmitter surges and to prepare the synapsis for a new neurotransmission event (3). In parallel, the energy demands of these metabolic movements must be fulfilled from substrates, such as glucose and lactate, obtained from cerebrovascular supplies (4, 5). Surrounding astrocytes coordinate all these tasks, playing a central role during neurotransmission, many times operating intercellularly as astrocytic networks (6, 7). Summarizing, the adequate operation of neurotransmission requires the transcellular coupling of neuronal and astrocytic metabolisms to a suitable supply of metabolic substrates from the microvasculature. Pathological alterations in these processes underlie the most morbid and prevalent neurological disorders, including ischemic or traumatic episodes and neurodegeneration.

Despite enormous progress in our understanding of neurotransmission during the last decades, important questions remain insufficiently explored including the quantitative assessment of transcellular cycles of glutamate, glutamine, and GABA supporting glutamatergic or gabaergic neurotransmissions, the preferred metabolic substrates, as glucose and/or lactate, supporting the energy demands under resting or stimulated conditions, and the mechanisms underlying neurovascular coupling. In addition, the important question on how all these processes occur and integrate under the *in vivo* situation reaches, in this context, vital relevance.

Recently, a variety of non-invasive approaches have allowed the investigation of these aspects *in vivo* (8), outstandingly, those involving functional magnetic resonance imaging and ^13^C magnetic resonance spectroscopy methods. The following e-book entitled “Transcellular Cycles Underlying Neurotransmission” provides an authoritative overview of these issues, compiling contributions from leading scientists in this field.

In the study of neuroglial interactions *in vivo*, Rodrigues et al. (9) provide a convenient introduction to the fundamentals of ^13^C NMR spectroscopy and its applications to cerebral energy metabolism, Duarte et al. (10) report on the compartmentalized metabolism of (1,6-^13^C_2_) glucose in the brain *in vivo*, Shen (11) reviews the mathematical modeling strategies used to simulate quantitatively the operation glutamate–glutamine cycle *in vivo*, and Sampol et al. (12) address the metabolism of glucose and lactate in the stimulated, awake, rat brain. Similarly, Bartnick-Olson et al. (13) illustrate the use of ^13^C NMR to evaluate the altered neuroglial interactions in response to traumatic brain injury.

The neurophysiological, metabolic, and cellular compartmentation events underlying functional neuroimaging by MRI are discussed by Moreno et al. (14), while Lizarbe et al. (15) cover the use of different MRI and MRS strategies to evaluate the ionic responses during hypothalamic activation by appetite stimulation. The role of astrocytic metabolic networks in metabolic coupling is discussed by Escartin and Rouach (16), whereas Bergessen and Gjedde (17) elaborate on the interesting hypothesis of lactate becoming a volume transmitter of metabolic states through the brain.

In summary, this e-book provides a broad coverage of recent progress in neuroglial coupling mechanisms underlying neuronal firing under physiological or pathological situations and their integration within astrocytic networks and associated neurovascular responses, as observed by advanced magnetic resonance imaging and spectroscopy methods *in vivo*.

We hope that this compilation becomes useful for a wide range of neuroscientists, from young students entering the field and looking for a global perspective, to established scientists, searching for specialized views on critical issues of cerebral neurotransmission *in vivo*.

## Conflict of Interest Statement

The authors declare that the research was conducted in the absence of any commercial or financial relationships that could be construed as a potential conflict of interest. The handling editor, Pierre J. Magistretti, declares that, despite being affiliated to the same institution as author, Blanca Lizarbe, the review was handled objectively and no conflict of interest exists.
